# Direct replacement of oral sodium benzoate with glycerol phenylbutyrate in children with urea cycle disorders

**DOI:** 10.1002/jmd2.12274

**Published:** 2022-02-02

**Authors:** Mildrid Yeo, Preeya Rehsi, Megan Dorman, Stephanie Grunewald, Julien Baruteau, Anupam Chakrapani, Emma Footitt, Helen Prunty, Melanie McSweeney

**Affiliations:** ^1^ Department of Paediatric Inherited Metabolic Disease Great Ormond Street Hospital NHS Foundation Trust and Institute for Child Health London UK

**Keywords:** glycerol phenylbutyrate, hyperammonaemia, liquid sodium benzoate, urea cycle disorders

## Abstract

Long‐term management of urea cycle disorders (UCDs) often involves unlicensed oral sodium benzoate (NaBz) which has a high volume and unpleasant taste. A more palatable treatment is licenced and available (glycerol phenylbutyrate [GPB], Ravicti) but guidance on how to transition patients from NaBz is lacking. A retrospective analysis of clinical and biochemical data was performed for eight children who transitioned from treatment with a single ammonia scavenger, NaBz, to GPB at a single metabolic centre; UCDs included arginosuccinic aciduria (ASA) (*n* = 5), citrullinaemia type 1 (*n* = 2) and carbamoyl phosphate synthetase I deficiency (CPS1) (*n* = 1). Patients transitioned either by gradual transition over 1–2 weeks (*n* = 3) or direct replacement of NaBz with GPB (*n* = 5). Median initial dose of GPB was 8.5 mL/m^2^/day based on published product information; doses were revisited subsequently in clinic and titrated individually (range 4.5–11 mL/m^2^/day). Pre‐transition and post‐transition mean ammonia levels were 37 μmol/L (SD 28 μmol/L) and 29 μmol/L (SD 22 μmol/L), respectively (*p* = 0.09), and mean glutamine levels were 664 μmol/L (SD 225 μmol/L) and 598 μmol/L (SD 185 μmol/L), respectively (*p* = 0.24). There were no reductions in levels of branched chain amino acids. No related adverse drug reactions were reported. Patients preferred GPB because of its lower volume and greater palatability. Direct replacement of NaBz with GPB maintained metabolic control and was simple for the health service and patients to manage. A more cautious approach with additional monitoring would be warranted in brittle patients and patients whose ammonia levels are difficult to control.

**Synopsis**

In stable well‐controlled patients, simple direct replacement of NaBz with GPB maintained metabolic control and was simple for the health service and patients to manage.

## 1 INTRODUCTION

The urea cycle is a metabolic pathway for the disposal of waste nitrogen via the conversion of ammonia to urea which is then excreted in the urine. Deficiencies of the enzymes or transporters responsible for converting ammonia to urea can result in the accumulation of toxic levels of ammonia in the blood and brain. The resulting encephalopathy can cause death or neurological and mental impairment.

Long‐term treatment of urea cycle disorders (UCDs) requires a combination of low‐protein diet, supplements of essential amino acids and other nutrients, medicines that control ammonia levels, and an emergency regimen for use during illness. The regime is challenging for patients and families because of the poor palatability, volume, and frequency of many of the treatments.^1^ The therapeutic goal is to prevent the irreversible toxicity caused by exposure of the brain to high levels of ammonia and thereby enable normal development and growth.^2^

One medicine used to control ammonia levels is oral sodium benzoate (NaBz), manufactured in the UK as a ‘special’. Specials are unlicensed medicines which are prescribed when a patient needs a specific formulation of a drug, which is not available as a licenced medicine. Although NaBz has been considered a mainstay of pharmacological therapy in chronic management of UCDs, some patients struggle to adhere to treatment because of its relatively large volume and poor palatability. A more recent introduction is Ravicti (1.1 g/mL glycerol phenylbutyrate [GPB]), which received its marketing authorisation in the US in 2013 and in the EU in 2015, and which is presented as a tasteless and odourless liquid, free from sugar and sodium, and in a much smaller volume than NaBz.^3^

Evidence supporting GPB as a well‐tolerated and effective medicine for UCD patients as young as 2 months of age is growing.^4^, ^5^ Since GPB became available in the UK in 2018, eight patients at our centre have transitioned to GPB from NaBz. All patients were on a single agent to control their ammonia levels. We present details of our approach to the transition and how it evolved over time.

## 2 METHODS

### 2.1 Data collection

A retrospective analysis of clinical and biochemical data was performed for UCD patients who were transitioned to GPB from single‐agent ammonia‐scavenging therapy with NaBz. All patients were managed by a single centre (Great Ormond Street Hospital, UK). Clinical data were reviewed from electronic medical records.

Data were collected for the period January 2019 to April 2021 and included diagnosis, age at diagnosis or presentation, reason for transition from NaBz to GPB, age at transition, method of transition, dose of NaBz pre‐transition, initial dose of GPB post‐transition, titrated dose of GPB post‐transition, plasma ammonia, glutamine, and branched chain amino acid levels pre‐transition and post‐transition, liver function pre‐transition and post‐transition, follow‐up duration, adverse events, frequency of metabolic decompensations since transition, most recent dose of GPB, and patient feedback.

The dose of NaBz pre‐transition was the latest dose that the patient was receiving immediately before the transition to GPB. The initial dose of GPB post‐transition was the dose of GPB that the patient transitioned to from NaBz. The titrated dose of GPB post‐transition was the adjusted dose that the patient was prescribed following their first routine clinic review post‐transition.

Ammonia was measured by reflectance absorbance spectrophotometry at 600 nm after reaction with a specific indicator (bromophenol blue) using the VITROS 5600 Integrated System (Ortho Clinical Diagnostics). Plasma amino acids were measured by absorption spectrometry at 570 nm of the ninhydrin derivative following protein precipitation with 5‐sulphosalicylic acid containing a specified quantity of internal standard (S‐(2‐Aminoethyl)‐L‐cysteine hydrochloride [AEC]) and separation by cation exchange chromatography using the Biochrom 30+ Amino Acid Analyser Physiological System (Biochrom Limited).

Ethical approval was not required as this was a retrospective study.

### 2.2 Daily dose of GPB

In all patients except Patient 1, the initial daily dose of GPB post‐transition was that recommended by the manufacturer for phenyl‐butyrate‐naïve patients: 8.5 mL/m^2^/day (9.4 g/m^2^/day) in patients with a body surface area (BSA) < 1.3 m^2^, or 7 mL/m^2^/day (8 g/m^2^/day) in patients with a BSA ≥ 1.3 m^2^ (Ravicti Summary of Product Characteristics [SPC], available at medicines.org.uk). Once patients had been transitioned to GPB and their ammonia levels were confirmed to be under control, doses were titrated at a routine clinic appointment. The titrated daily dose of GPB was based on the patient's protein tolerance and required daily dietary protein intake, and also took into account their previous NaBz requirements.

### 2.3 Method of transitioning from NaBz to GPB

Transitioning to GPB was initiated only if ammonia levels were normal and patients were clinically well. The approach to transitioning evolved as clinical experience with the process grew. The initial approach for the first three patients involved a gradual phased transition, the latter approach was direct replacement of NaBz with GPB. The gradual transition took place over 1 or 2 weeks and involved three phases: (1) patients were initiated on half the target daily dose of GPB in addition to their normal NaBz dose, (2) patients increased their GPB dose to the full target daily dosage and halved their normal NaBz dose, and (3) NaBz was stopped completely, and patients continued with the full target daily dose of GPB.

Ammonia, glutamine, and branched chain amino acid levels were monitored before, during, and after transition. In addition, liver function tests were measured before transition and once the patient was established on the titrated daily dose of GPB.

### 2.4 Statistical analysis

Statistical analysis was undertaken using the Microsoft Excel Analysis ToolPak. Three measurements of ammonia were below the limit of detection (<9 μmol/L) so a pragmatic approach was taken and a measurement of 8 μmol/L used as a substitute for the actual unknown value; this applied to one measurement pre‐transition and two measurements post‐transition. A paired *t*‐test was used to analyse changes in ammonia, glutamine, and branched chain amino acid levels before and after transition. Exploratory regression analysis was undertaken to assess the linear associations between ammonia and glutamine levels pre‐transition and post‐transition. Exploratory analysis of the relationship between the dose of NaBz pre‐transition and the titrated dose of GPB post‐transition was also undertaken. A *p* value <0.05 was considered statistically significant.

## 3 RESULTS

### 3.1 Demographics

Eight patients with UCDs (seven males and one female) were identified from eight families. Five patients had arginosuccinic aciduria (ASA), two patients had citrullinaemia type 1 and one patient had carbamoyl phosphate synthetase I deficiency (CPS1). Six patients were diagnosed in the neonatal period (range 1–15 days) (four with ASA and two with citrullinemia type 1), one patient was diagnosed at age 1 year (ASA) and another at 2 years (CPS1).

Patients were transitioned from NaBz to GPB between January 2019 and March 2021. The post‐transition data collection period spanned more than 2 years, and a total of 10 patient‐years of data were collected (Table 1). Patients received GPB for a median of 18 months (range 1–25). Reasons for the transitions were related to the risk of poor treatment adherence and associated clinical sequelae as a result of poor tolerability/palatability of NaBz.

**TABLE 1:** Demographics and dose

Patient	Diagnosis	Age at diagnosis	Age at transition (years)	Transition approach	Dose of NaBz pre‐transition (mg/kg/day)	Initial dose of GPB post‐transition (mL/m^2^/day)	Titrated dose of GPB post‐transition (mL/m^2^/day)	Follow‐up duration (months)
1	CPS1	17 months	4.8	Gradual	165	11.0	Not titrated	26
2	ASA	Day 4	5.8	Gradual	300	8.5	8.5	25
3	ASA	Day 3	7.1	Gradual	180	8.5	7.2	23
4	ASA	Day 5	15.4	Direct	176	7.0	6.5	21
5	ASA	12 months	9.0	Direct	91	8.5	4.5	12
6	Citrullinaemia	Day 1	0.23	Direct	253	8.5	8.5	8
7	Citrullinaemia	Day 15	2.9	Direct	305	8.5	8.5	4
8	ASA	Day 5	0.08	Direct	350	8.5	8.5	1

Abbreviations: ASA, argininosuccinic acidemia, or argininosuccinate lyase deficiency; citrullinaemia, argininosuccinate synthetase deficiency type 1; CPS1, carbamoyl phosphate synthetase I deficiency; GPB, glycerol phenylbutyrate; NaBz, sodium benzoate.

### 3.2 Method of transition

Patients 1, 2, and 3 underwent a gradual transition from NaBz to GPB. Patients 4, 5, 6, 7, and 8 underwent a direct replacement of NaBz with GPB.

### 3.3 Doses of NaBz and GPB

Pre‐transition, the mean dose of NaBz was 228 mg/kg/day (SD 88 mg/kg/day); mean doses for the gradual transition and direct replacement approaches were 215 mg/kg/day (SD 74 mg/kg/day) and 235 mg/kg/day (SD 103 mg/kg/day), respectively.

The initial dose of GPB post‐transition for Patient 1, who was the first patient to transition, was 11 mL/m^2^/day. All subsequent patients were transitioned to the initial dose of GPB recommended by the manufacturer (Immedica Pharma AB) for phenyl‐butyrate‐naive patients, which is based on body surface area; it was 8.5 mL/m^2^/day for Patients 2, 3, 5, 6, 7, and 8, and 7 mL/m^2^/day for Patient 4.

If warranted, dose was titrated at a patient's first routine clinic appointment post‐transition: the mean titrated dose of GPB for Patients 2 to 8 was 7.5 mL/m^2^/day (SD 1.53 mL/m^2^/day); mean titrated doses for the gradual transition and direct replacement approaches were 7.9 mL/m^2^/day (SD 0.92 mL/m^2^/day) and 7.3 mL/m^2^/day (SD 1.79 mL/m^2^/day), respectively. The outcome of titrating the doses of GPB in Patients 2–8 was that four doses were left unchanged, and three were decreased.

**FIGURE 1:**
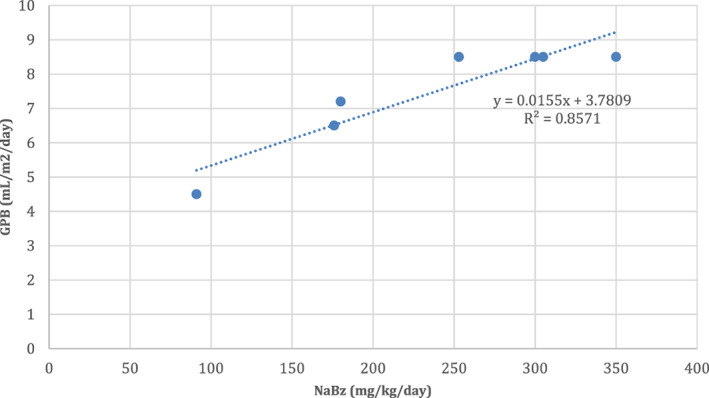
Association between NaBz dose pre‐transition and titrated GPB dose post‐transition. The chart features information for Patients 2–8. Patient 1 is excluded from this chart because their dose of GPB was not titrated after transition to GPB. GPB, glycerol phenylbutyrate; NaBz, sodium benzoate

Excluding Patient 1, who received a dose of 11 mL/m^2^/day GPB despite being on a relatively moderate dose of NaBz, there was an association (*R* ^2^ = 0.86, *p* = 0.003) between the dose of NaBz pre‐transition and the titrated dose of GPB post‐transition; a retrospective analysis found that the titrated dose of GPB was predicted by the formula: GPB (mL/m^2^/day) = 0.0155 × NaBz (mg/kg/day) + 3.7809 (Figure 1). The doses calculated using this formula were between 91 and 115% of the titrated doses administered.

Patients received GPB orally three times a day with meals, although this was adjusted to twice daily or four times daily in some patients based on patient preference.

### 3.4 Ammonia levels

Pre‐transition levels of ammonia were measured within 3 weeks prior to transition. The first post‐transition ammonia levels were measured a median of 12 days after transition (range 1 to 29 days). Subsequent post‐transition levels were measured every few months in line with usual clinical practice.

**FIGURE 2:**
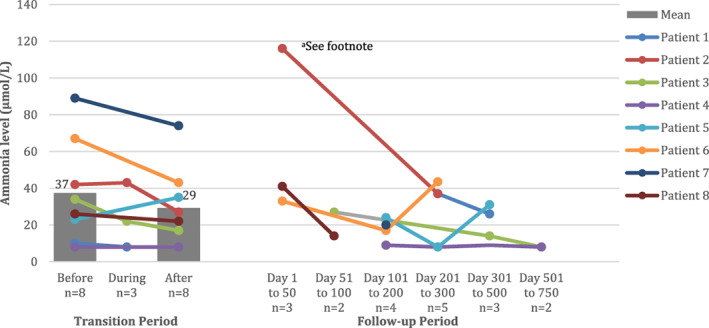
Ammonia levels during the transition and follow‐up periods. Patients 1, 2, and 3 underwent a gradual transition from NaBz to GPB. Patients 4, 5, 6, 7, and 8 underwent a direct transition. Ammonia levels were measured before, during, and after transition and at subsequent follow‐up visits. A solid circle indicates a measurement. The grey bars and associated data labels indicate mean values and are presented before and after transition only, when measurements were available for all eight patients. ^a^One metabolic decompensation occurred as a result of illness; the ammonia level for Patient 2 reached 116 μmol/L, but the patient was able to revert to oral treatment with GPB after 24 hours following the administration of intravenous sodium phenylbutyrate, arginine, a saline bolus and fluids. GPB, glycerol phenylbutyrate; NaBz, sodium benzoate

Mean ammonia levels pre‐transition and post‐transition were 37 μmol/L (SD 28 μmol/L) and 29 μmol/L (SD 22 μmol/L) (*p* = 0.09) (Figure 2). There was an association between ammonia levels pre‐transition and‐post‐transition (*R* ^2^ = 0.85, *p* = 0.001).

For the gradual transition approach, mean ammonia levels pre‐transition and post‐transition were 29 μmol/L (SD 17 μmol/L) and 17 μmol/L (SD 10 μmol/L), respectively (*p* = 0.14). For the direct replacement approach, mean ammonia levels pre‐transition and post‐transition were 43 μmol/L (SD 34 μmol/L) and 36 μmol/L (SD 25 μmol/L), respectively (*p* = 0.37).

Pre‐transition, two patients had an ammonia level above 50 μmol/L; these were Patients 6 and 7 with ammonia levels of 67 and 89 μmol/L, respectively. Post‐transition, Patient 7 remained above 50 μmol/L, with an ammonia level of 74 μmol/L.

### 3.5 Glutamine, branched chain amino acids, and liver function

Pre‐transition levels of amino acids were measured within 3 weeks prior to transition except for Patient 7, whose amino acid levels were measured 98 days prior to the start of transition. The first post‐transition amino acid levels were measured a median of 12 days after transition (range 1–29 days) except for Patient 5, whose amino acid levels were measured 198 days after transition. Subsequent post‐transition levels were measured every few months in line with usual clinical practice.

**FIGURE 3:**
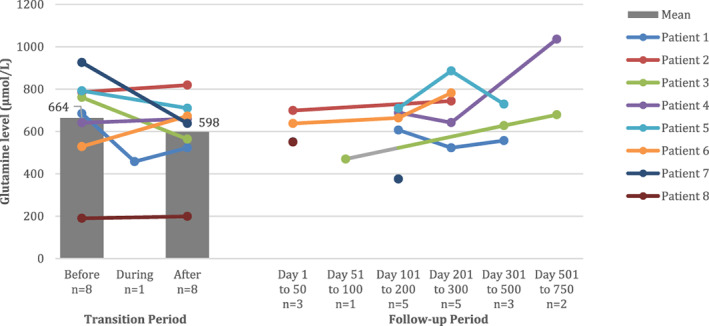
Glutamine levels during the transition and follow‐up periods. Patients 1, 2, and 3 underwent a gradual transition from NaBz to GPB. Patients 4, 5, 6, 7, and 8 underwent a direct transition. Glutamine levels were measured before, during, and after transition and at subsequent follow‐up visits. A solid circle indicates a measurement. The grey bars and associated data labels indicate mean values and are presented before and after transition only, when measurements were available for all eight patients. Glutamine levels during the transition were available for Patient 1 only. GPB, glycerol phenylbutyrate; NaBz, sodium benzoate

Glutamine levels were broadly unchanged by the transition (Figure 3). Mean glutamine levels pre‐ and post‐transition were 664 μmol/L (SD 225 μmol/L) and 598 μmol/L (SD 185 μmol/L), respectively (*p* = 0.24). There was an association between glutamine levels pre‐ and post‐transition (R^2^ = 0.60, *p* = 0.02).

For the gradual transition approach, mean glutamine levels pre‐ and post‐transition were 744 μmol/L (SD 53 μmol/L) and 635 μmol/L (SD 160 μmol/L), respectively (*p* = 0.27). For the direct replacement approach, mean glutamine levels pre‐ and post‐transition were 615 μmol/L (SD 281 μmol/L) and 576 μmol/L (SD 212 μmol/L), respectively (*p* = 0.61).

There was no significant association between glutamine and ammonia levels, neither before nor after transition.

There were no significant reductions in the levels of branched chain amino acids over the course of the transition and when measured subsequently.

Pre‐transition, Patients 2, 3, and 8 had deranged liver function, with alanine aminotransferase (ALT) levels of 108, 84, and 71 μmol/L. All other patients had normal liver function. Post‐transition, liver function in Patients 3 and 8 had normalised with ALT levels of 30 and 38 μmol/L, respectively. Liver function in Patient 2 was less deranged, with an ALT level of 76 μmol/L.

### 3.6 Protein tolerance

Dietary protein tolerance was unchanged throughout the course of the study. Dietary protein allowance was adjusted in line with usual practice, with target protein intake based on World Health Organisation (WHO) recommendations.^6^

### 3.7 Safety and tolerability

During the cumulative 10 patient‐years of treatment with GPB there have been no reports of related adverse drug reactions. One metabolic decompensation occurred as a result of illness; the ammonia level for Patient 2 reached 116 μmol/L. Intravenous sodium phenylbutyrate, arginine, a saline bolus, and fluids were administered, and the patient was able to revert to oral treatment with GPB after 24 h.

All patients and/or their families provided unsolicited informal feedback that they preferred GPB to NaBz for one or more reasons of its lower volume, greater palatability, and easier administration.

## 4 DISCUSSION

The aims of long‐term management of UCDs are to maintain stable metabolic control, eliminate chronic complications, and achieve normal development and growth.^1^ A key aspect is controlling ammonia levels, which requires the provision of effective treatments and adherence to those treatments by patients.

The target ammonia level we aim for outside the neonatal period is <50 μmol/L. In our case series, the mean ammonia levels pre‐transition were 37 μmol/L (SD 28 μmol/L) and post‐transition 29 μmol/L (SD 22 μmol/L). Pre‐transition, two patients had an ammonia level above the target of 50 μmol/L. Post‐transition, one patient was above the target level. The dose of NaBz or GPB required to achieve a satisfactory ammonia level varied between patients, and there was no apparent association between dose of NaBz or GPB and ammonia level; this illustrates the importance of individualising treatment. Ammonia control was regarded as maintained in all patients during the transition from NaBz to GPB regardless of mode of transition.

When we transitioned our first three patients from NaBz to GPB we adopted a gradual approach. Building on that experience, we transitioned the next five patients by a direct replacement of GPB for NaBz. We found that this direct transition maintained ammonia control and meant none of the complexity for the health system and patients of a gradual transition. Having the option to directly replace NaBz with GPB while monitoring biochemical markers allowed us to simplify and accelerate the process, saving time and reducing risk.

One challenge when transitioning patients from NaBz to GPB was determining the appropriate dose of GPB. The SPC for GPB features recommended doses for patients who are (1) phenylbutyrate (PBA)‐naive, (2) transitioning from sodium phenylbutyrate (NaPBA), and (3) transitioning from NaPBA/NaBz injection.^3^ However, the SPC does not feature recommended doses for patients transitioning from NaBz. Technically, our patients were PBA‐naïve, so the initial dose of GPB we used in all patients except Patient 1 was that recommended in the SPC. When we saw a patient at their routine appointment post‐transition, we reviewed their dose and titrated it based on the patient's protein tolerance and required daily dietary protein intake. We also took into account their previous NaBz requirements. Looking back at the association between the dose of NaBz and titrated dose of GPB, the titrated dose was quite well predicted from the pre‐transition dose of NaBz using the formula: GPB (mL/m^2^/day) = 0.0155 × NaBz (mg/kg/day) + 3.7809. Metabolic control was maintained in all patients receiving a titrated dose, leading us to consider adopting this formula when determining GPB dose in future patients who are stable and well controlled but transitioning from oral NaBz. Using the formula may result in an initial dose of GPB closer to a patient's needs, more so than the approach of starting all patients on the same dose. Patient 5, for example, was controlled on a dose of NaBz of 91 mg/kg/day, then was transitioned to an initial dose of GPB of 8.5 mL/m^2^/day, before being titrated down to a dose of GPB of 4.5 mL/m^2^/day.

Treatment with GPB was welcomed by patients and/or their families. Proactive, unsolicited feedback was that GPB was preferred to NaBz for one or more reasons of its lower volume, greater palatability, and easier administration. Similar findings were reported in a study which explored the effects of the transition from NaBz or NaPBA to GPB on health‐related quality of life of nine children with a UCD and their carers; the authors reported that reluctance to take NaBz or NaPBA was often attributed to elements such as poor taste, burden of treatment and subsequent nausea experienced after the medication had been administered.^7^ The authors suggest that improved taste, timing, and administration of GPB appeared to contribute towards greater medication adherence, and that relying on GPB as primary pharmacological treatment could reduce the ‘battle’ of administering medication. For patients with UCDs, non‐adherence can have significant clinical consequences therefore an improvement in adherence could result in improved outcomes.^8^

In our cohort, GPB appeared to be well‐tolerated. There were no reported drug‐related adverse events in over 10 patient‐years of treatment. One episode of hyperammonaemia occurred but this was related to illness, not GPB. Liver function was observed to have improved in some patients, which could be a reflection of better metabolic stability. Glutamine levels were broadly unchanged, with all but one measurement below the 1000 μmol/L target recommended for the long‐term management of patients with UCDs.^1^ The mean glutamine level in patients at the end of the transition period to GPB was 598 μmol/L (SD 185 μmol/L) which is in line with other studies in children transitioned to GPB.^9^, ^10^ However, those studies found a trend towards a decrease in glutamine with long‐term use of GPB which was not evident in our study, possibly because of our small sample size and the real‐world (uncontrolled) nature of our data.

Levels of branched chain amino acids were maintained after transition from NaBz to GPB. It has been reported previously that PBA causes a reduction in levels of branched chain amino acids, although this conclusion was drawn from a study of patients receiving NaPBA rather than GPB.^11^ Studies evaluating the use of GPB in paediatric patients found that long‐term use was associated with normal levels of glutamine and branched chain amino acids.^4^, ^5^, ^9^, ^10^ The reason for this observed difference between NaPBA and GPB has not been elucidated but presumably relates to changes in the metabolism of PBA conferred by the glycerol moiety of GPB. A population pharmacokinetic model using data from patients exposed to NaPBA and GPB in crossover studies found that administration of GPB was associated with slower PBA absorption and greater pre‐systemic conversion of PBA to phenylacetic acid (PAA) and/or phenylacetylglutamine (PAGN).^12^ A crossover study in patients with UCDs compared the ammonia control and pharmacokinetics of NaPBA versus GPB; ammonia levels were 30% lower with GPB than with NaPBA, and systemic exposure to PBA was 27% lower with GPB than with NaPBA.^13^ While these details are not offered as an explanation of why levels of branched chain amino acids in patients receiving long‐term treatment with GPB have been found to be normal, it does demonstrate that NaPBA and GPB are metabolised differently.

Overall, GPB is an effective licenced alternative to NaBz. Its use is in line with the guidance that children should be able to receive medicines that are safe, effective, appropriate for their condition, palatable, and available with minimal clinical risk.^14^ The fact that GPB is licenced and supported by a published evidence base avoids the clinical and ethical dilemmas associated with prescribing an unlicensed medicine, and it means that patients are better able to participate in the shared decision‐making process.^15^ Transitioning patients from NaBz to GPB has been shown to improve acceptability, which might translate into improved adherence and tighter control of ammonia levels. This is important, because even slightly raised levels can cause clinical sequelae if left unchecked in the long‐term. This can be a challenge with NaBz because increasing the dose increases the issues associated with volume, sodium, and palatability. With GPB, dose escalation is simpler because it is nearly tasteless and odourless, contains no sugar or sodium, and is administered in volumes that are relatively low compared with other ammonia scavengers. In Europe, the GPB licence does not feature an upper dose limit, meaning treatment can be titrated to achieve tight control and might avoid the need to add in a second ammonia scavenger.

The inherent limitations of our evaluation of the safety and effectiveness of a direct transition from NaBz to GPB are acknowledged: the number of patients included is small, there was no control group, data were collected retrospectively, samples for biochemistry analysis were uncontrolled, and the patients selected for transition were receiving a single ammonia scavenger to control ammonia levels which meant that those patients receiving more than one ammonia scavenger, often those with the most common type of UCD, ornithine transcarbamylase (OTC), were not considered in scope for transition. It should be emphasised that the patients selected for transition were on a single agent to treat their UCD, and that there may be some children in whom a more cautious approach is warranted – those who are more fragile and susceptible to metabolic decompensation, for example. Nonetheless, in the absence of any controlled clinical trial data, the information we have gathered is needed to help inform clinical practice. The results have provided our clinical team with confidence to transition more patients from NaBz to GPB by direct replacement.

## 5 CONCLUSION

Transitioning from NaBz to GPB was effective, well tolerated by patients, and maintained metabolic control. Direct replacement was favoured over gradual transition because it meant simpler prescribing for the health service, and simpler instructions for patients to follow. The lower volume, greater palatability, and easier administration reported by patients in relation to GPB has the potential to improve adherence, which may in turn improve outcomes. A more cautious approach with additional monitoring would be warranted in patients on multiple treatments, brittle patients, and those patients whose ammonia levels are difficult to control.

## CONFLICT OF INTEREST

The authors declare no conflicts of interest.

## INFORMED CONSENT

The legal representatives of the children described in this case report provided consent for publication of the data.
